# Supplementary figures and images for: Negligible-cost microfluidic device fabrication using 3D-printed interconnecting channel scaffolds
Source: PLoS One. 2021 Feb 3;16(2):e0245206. doi: 10.1371/journal.pone.0245206 (PMC7857642; doi:10.1371/journal.pone.0245206)

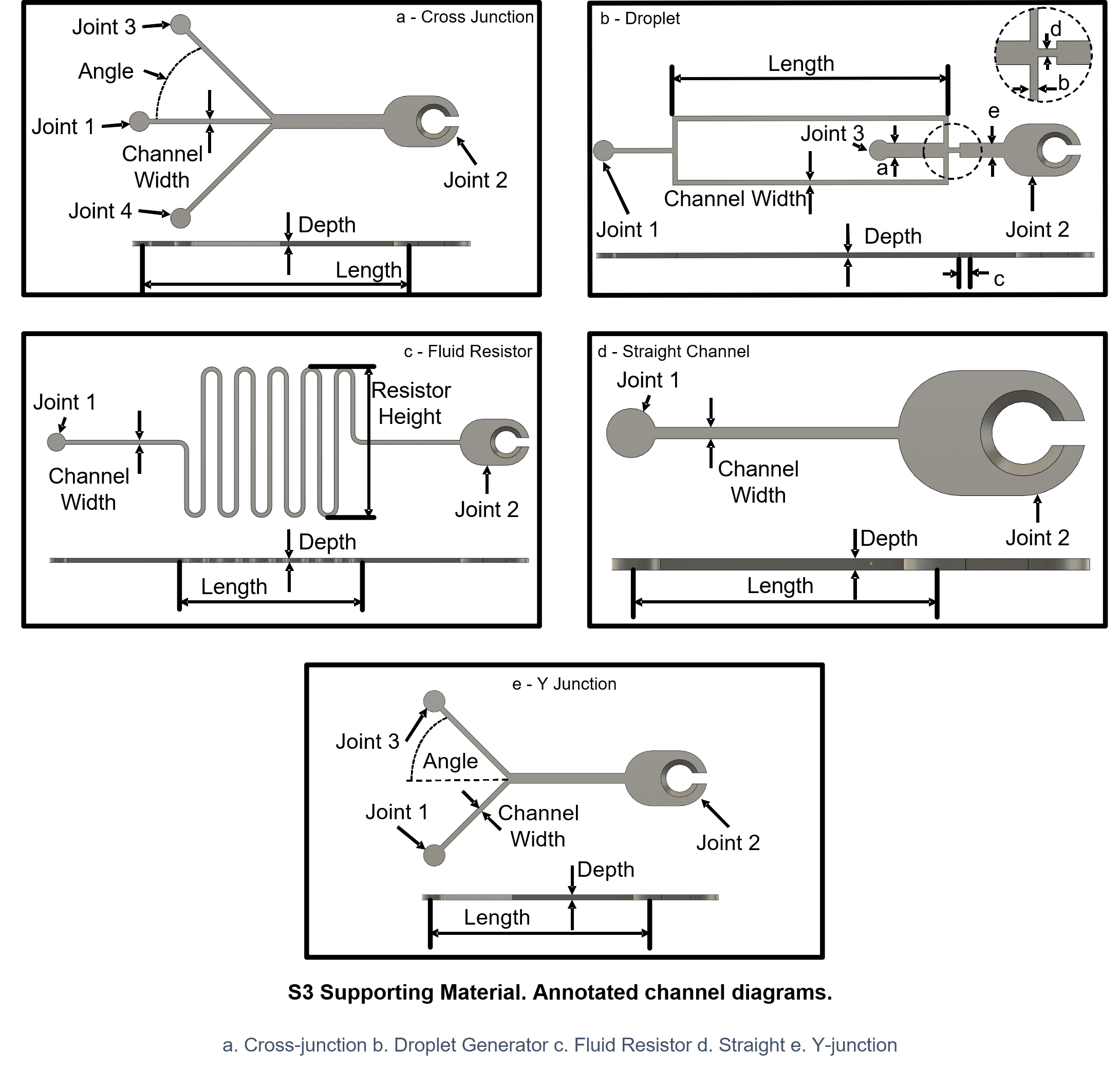

Supplement: S1 Fig — a. Cross-junction b. Droplet Generator c. Fluid Resistor d. Straight e. Y-junction. (TIF) [file pone.0245206.s001.tif]

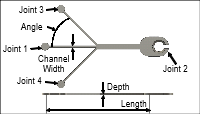

Supplement: S1 Supporting information — (ZIP) [file pone.0245206.s003.zip › MicroChannels/resources/images/cross_junction.png]

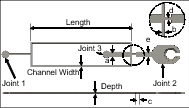

Supplement: S1 Supporting information — (ZIP) [file pone.0245206.s003.zip › MicroChannels/resources/images/droplet.png]

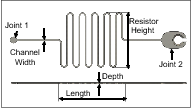

Supplement: S1 Supporting information — (ZIP) [file pone.0245206.s003.zip › MicroChannels/resources/images/resistor.png]

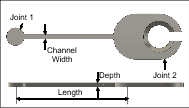

Supplement: S1 Supporting information — (ZIP) [file pone.0245206.s003.zip › MicroChannels/resources/images/straight.png]

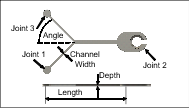

Supplement: S1 Supporting information — (ZIP) [file pone.0245206.s003.zip › MicroChannels/resources/images/y_junction.png]

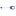

Supplement: S1 Supporting information — (ZIP) [file pone.0245206.s003.zip › MicroChannels/resources/logos/16x16.png]

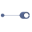

Supplement: S1 Supporting information — (ZIP) [file pone.0245206.s003.zip › MicroChannels/resources/logos/16x16@2x.png]

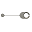

Supplement: S1 Supporting information — (ZIP) [file pone.0245206.s003.zip › MicroChannels/resources/logos/16x16@2x-disabled.png]

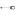

Supplement: S1 Supporting information — (ZIP) [file pone.0245206.s003.zip › MicroChannels/resources/logos/16x16-disabled.png]

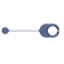

Supplement: S1 Supporting information — (ZIP) [file pone.0245206.s003.zip › MicroChannels/resources/logos/32x32@2x.png]

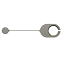

Supplement: S1 Supporting information — (ZIP) [file pone.0245206.s003.zip › MicroChannels/resources/logos/32x32@2x-disabled.png]

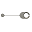

Supplement: S1 Supporting information — (ZIP) [file pone.0245206.s003.zip › MicroChannels/resources/logos/32x32-disabled.png]
