# Supplementary material for: Negligible-cost microfluidic device fabrication using 3D-printed interconnecting channel scaffolds
Source: PLoS One. 2021 Feb 3;16(2):e0245206. doi: 10.1371/journal.pone.0245206 (PMC7857642; doi:10.1371/journal.pone.0245206)
Supplement: Attachment — Submitted filename: Response to Reviewers.docx [file pone.0245206.s010.docx]

Reviewer #1: Thank the authors for the revision of this manuscript. The authors have addressed my previous querries and concerns. I therefore recommand publication in Plos One.

Thank you very much for your feedback and your constructive comments. They were greatly appreciated.

Reviewer #2: The authors have made significant improvements in the revised version of the manuscript. Most of my concerns have alleviated. The 3d-printing-based microfluidic device fabrication method introduced in the paper substantially lowers the barrier for undergraduate students and other industry practitioners with low cost and easy-to-implement processes. The authors elaborate on all the 3d modeling, printer setting, and fabrication parameters. Before considering for publication, some minor issues are expected to be resolved:

We would like to thank the reviewer for their comments and attention to detail. We have corrected these issues.

1. Page 2, Figure 1, I cannot find figure 1(g), which is introduced in the caption of Figure 1.

Thank you very much for noticing this. This has been corrected.
2. Table 1, the unit of the build plate temperature is wrong.

Again, we thank you for pointing this out. This has been corrected.
3. Make sure all the literature is correctly referenced (e.g., Line 213).

Thank you. We have corrected this issue.
